# Women’s Knowledge, Attitude and Practice on Cervical Cancer and Its Screening in Dhaka, Bangladesh

**DOI:** 10.31557/APJCP.2021.22.10.3327

**Published:** 2021-10

**Authors:** M.d Omar Qayum, Mallick Masum Billah, Rehena Akhter, Meerjady Sabrina Flora

**Affiliations:** 1 *Institute of Epidemiology, Disease Control and Research (IEDCR), Bangladesh. *; 2 *Bangabandhu Sheikh Mujib Medical University (BSMMU), Bangladesh. *; 3 *Additional Director General (Planning and Development), Directorate General of Health Services, Bangladesh. *

**Keywords:** Cervical cancer, screening, VIA test, HPV- women, Bangladesh

## Abstract

**Background:**

Cervical cancer is the fourth most common cancer among women in the world. Visual Inspection with Acetic Acid (VIA) is a common screening test for cervical cancer in Bangladesh. This study will assess the knowledge, attitude and practice towards cervical cancer and screening among women residing in Dhaka district.

**Methods:**

A cross-sectional survey was conducted among 956 women aged 30 years and above in Dhaka. The women’s score on knowledge, attitude and practice were categorized as sufficient or insufficient. We calculated frequencies and used binary logistic regression to describe and assess the association between scores and socio-demographic characteristics of respondents.

**Results:**

Most (87%) respondent knew about cervical cancer and 13% knew that HPV is a risk factor for cervical cancer. Women who had sufficient knowledge were more likely to test VIA than those who had insufficient knowledge (39%, OR: 2.5; CI: 1.6, 2.8). Most (92%) would advise other women to have a VIA test. However, only 26% had a VIA test and 2% were vaccinated in private health care facilities for Human Papilloma Virus (HPV). Women who had sufficient attitude were equally likely to test VIA than those who had insufficient attitude. The VIA was underutilized because of low privacy during examination, unaware that VIA screened for cervical cancer, belief that they must pay for the test, and nurses performed examination.

**Conclusion:**

Women were knowledgeable about cervical cancer and likely to have a VIA test. However, the VIA test in underutilized and HPV vaccine coverage was low.

## Introduction

Cervical cancer is the most common cancer in developing countries (Goel et al., 2005). In Bangladesh, cervical cancer is the second presiding cause of cancer death of women aged 15-44 years. Age-standardized incidence rates of cervical cancer were 10.6 per 100000 Bangladeshi women in 2018. According to International Agency for Research on Cancer an estimated 604000 women were diagnosed with cervical cancer in 2020 with 342000 death. About 5,214 cervical cancer deaths occur annually in Bangladesh (“HPV INFORMATION CENTRE,”n.d).

Insufficient screening, high prevalence of risk factors, early marriage, sexual intercourse at an early age, high parity, low socio-economic condition is some of the reasons for the high burden of cancer in Bangladesh (n.d.). The majority of cervical cancer could be prevented if all women were participated in a screening program (Papri et al., 2015). According to WHO, Bangladesh screening guidelines recommend screening from 30 years of age with population screening coverage of women aged 30 to 49 years every three years. 

With the assistance of Global Alliance for Vaccines and Immunizations (GAVI), Ministry of Health has introduced Human Papilloma Virus (HPV) vaccine in Bangladesh. According to WHO recommendations, girls are vaccinated with HPV vaccine between 9-13 years of age. In this regard Bangladesh piloted a school-based vaccination program that vaccinated 10 years old girls in primary schools in selected Upazilas. As part of the pilot, girls who cannot be vaccinated in school can get vaccinated through the routine EPI sites (“HPV vaccine introduced in Bangladesh,”n.d).

Smear cytology is the standard screening test for cervical cancer but it is challenging to implement in developing countries due to the unavailability of logistic pre-requisites and high-quality laboratories. In response to these challenges, more cost-effective alternative methods such as visual inspection are being tested for use in low-resource settings (Denny et al., 2006). Studies suggest that VIA is both sensitive and specific enough to reduce the incidence and mortality of cervical cancer in developing countries (Sankaranarayan et al., 2001). 

Early detection of cervical cancer by screening can prevent most cervical cancer cases. The Government of Bangladesh introduced screening services free of cost for all women of 30-60 years in all districts of Bangladesh (“Home-Bangladesh academy of Pathology (BAP),”n.d.). Awareness of the successful treatment magnitude of disease and possible warning signs of cervical cancer among the public is important for early identification and reduction of the burden of this disease. 

With this objective, the present study was conducted to assess the level of knowledge, attitude and practice regarding cervical cancer and VIA screening among women. 

## Materials and Methods

*Methods and sampling*

The present study was a cross-sectional KAP survey among women aged 30 years and above. Among the area of Bangladesh where VIA screening program was ongoing, we selected Keraniganj Upazila and Tejgaon area of Dhaka district randomly. In these two areas, cervical cancer screening programme was ongoing. We considered Tejgaon area as urban area and Keraniganj as semi urban area. Using the Cochran’s formula, we found a representative sample of 956 women for our study where 411 eligible women were from Tejgaon area and 545 women were from Keraniganj.

Our study population was recruited by going to every fifth household and identifying every woman 30 years of age and above in that household. If there was more than one eligible woman, one was randomly selected and interviewed after obtaining written informed consent. If there was no eligible woman in the fifth household, the team proceeded to the sixth household or the seventh or n^th ^until an eligible woman was recruited. After completion of the interview, the team repeated this sampling procedure. The study respected each respondent’s freedom to participate and adhered to all research principles pertaining to privacy and confidentiality. Written informed consent was sought from all the participants.

*Exclusion criteria*

We excluded women below 30 years of age, those who were not permanently residing in Tejgaon and Keraniganj area, those who were not interested in participating, or did not give consent to be interviewed.

*Data collection tools and analysis*

A pre-tested questionnaire based on the study objectives was used to collect data. A face-to-face interview was taken at home after getting informed written consent from the respondents. Continuous variable like age, educational status, monthly expenditure, age at first marriage, number of alive children and parity were transformed into categorical variables for ease of analysis. The main outcomes of interest of this study were score of knowledge, attitude, and practice.

We calculated descriptive statistics like frequency and percentage of socio demographic characteristics of target population and women’s score on knowledge, attitude, and practice of VIA test. We used a cut-off of greater or equal 11 to denote “sufficient score” on knowledge, greater or equal to 5 to denote “sufficient score” on attitude and greater or equal to 3 to define “sufficient score” on practice. To measure the association between women’s socio demographic characteristics and undergone VIA test we used binary logistic regression. The association between respondent’s score and socio demographic characteristics was also assessed by binary logistic regression. Stata 14.0 (Stata Corp. 2015. Stata Statistical Software: Release 14. College Station, TX: Stata Crop LP) was used to calculate frequencies and odds ratios.

## Results

*Socio demographic characteristics of the respondents regarding VIA screening*

Table 1 shows that a total of 956 women were enrolled in our study. Among the study population highest proportion (35%) of women belonged to the age group 31 to 35 years. More than half (57%) of the population pertained to semi urban area and 26% of women had undergone through VIA test.

Table 2 implies that from the eligible respondents, women of 41-45 years old were more likely to test VIA than the youngest group (37%, OR: 1.7; 95% CI: 1.1, 2.6). In semi urban area practicing of VIA test was lower than the urban area (24%, OR: 0.8; 95% CI: 0.6, 1.0). There was a strong association between VIA screening and the level of education. Women who were from secondary educational level were more likely to go under VIA test than the illiterate women (35%, OR: 1.8; 95% CI: 0.8, 4.0) which was not statistically significant. Our study findings showed that testing VIA was likely to increase gradually with the increase of women’s age at first marriage (43%; OR: 2.9, CI: 1.7, 4.9) and with richest wealth quintile (31%; OR: 1.9, CI: 1.2, 3.1)

Table 3 epidemiological studies showed that respondents had highest knowledge about cancer and cervical cancer and on the other hand they had lowest knowledge about Galactorrhea and sexually transmitted disease. Three top risk factors reported by respondents were early marriage, sexually transmitted disease, and unhygienic condition of cervix. Only 13% knew HPV as a risk factor for cervical cancer. Half of the respondents had knowledge of tests for CA cervix detection. Among the study population 26% had ever tested VIA and only 2% had ever received HPV vaccine. We observed that proper privacy was not maintained in most cases during VIA test.

Table 4 showed that there was a significant association between socio-demographic characteristics and score of knowledge about cervical cancer. Women with higher knowledge were of younger age group, married, higher education, employee, higher income, non-Muslim, older age at first marriage and low parity. The knowledge on cervical cancer was likely to increase among women of secondary education level (70%, OR: 5.02; CI: 2.19, 11.49) than the illiterate women and employed women (64%, OR: 2.15; CI: 0.98, 4.71) though it is statistically insignificant.

We found that sufficient score of attitudes towards VIA screening was likely to increase in employed and locals of semi urban area. Married women showed three times higher attitude towards VIA test than divorced, widowed or separated women (18%, OR: 0.3; CI: 0.2, 0.5). The likelihood of showing attitude towards VIA test increasesd as the age at first marriage increasesd (49%, OR: 1.9; CI: 1.1, 3.1).

In regards of practice, Table 4 showed that the women of 41-45 years old scored 1.8 times higher than the women of ≤ 35 years old (29%, OR:1.8; CL: 1.1, 2.8). The women who lived in semi urban area scored better than the women who lived in urban area (20%, OR: 1.3; CI: 0.9, 1.3). Employed women were more likely to obtain a sufficient score than the housewife (21%, OR: 1.2; CI: 0.5, 3.0) though it is not statistically significant. 

One of the main concerns of our study was to find out the influencing characteristics of VIA screening. Our study findings showed a significant relationship between sufficient score of knowledge and going under VIA screening. Women who had sufficient knowledge were more likely to test VIA than who had insufficient knowledge (39%, OR: 2.5; CI: 1.6, 2.8) (Table 5).

The main source of information regarding cervical cancer of the eligible women was friends and relatives (76%) and followed by television (50%). Regarding cervical cancer screening tests, most of them were informed by their friends and relatives or close acquaintances (60%), followed by doctors (50%) (Table 6).

**Table 1:** Socio Demographic Characteristics of Women, Keraniganj Upazila and Tejgaon area of Dhaka district, Bangladesh, 2017

Socio demographic characteristics	N=956	
	n	%
Age (Years)		
≤ 35	336	35
36-40	209	22
41-45	132	14
46-50	142	15
51-55	70	7
56-60	44	5
>60	23	2
Mean(±SD)	41.23(9.09)	
Median (IQR)	39 (34-47)	
Marital status		
Married	845	88
Others	111	12
Residence		
Urban	411	43
Semi Urban	545	57
Educational status		
Illiterate/only Signature	230	24
Primary	696	73
Secondary	30	3
Occupation		
Housewife	928	97
Employee	28	3
Monthly expenditure (Tk)		
≤ 15000	269	28
16,000-30,000	565	59
31,000-90,000	122	13
Mean (±SD)	22099.4 (10696.4)	
Median (IQR)	20000 (15000-25500)	
Religion		
Muslim	931	97
Non-Muslim	25	3
Age at first marriage (Years)		
≤15	395	41
16-20	383	40
21-25	102	11
≥26	76	8
Mean (±SD)	17.5 (4.8)	
Median (IQR)	16 (14-20)	
Number of alive children		
0	13	1
1-2	409	43
3-5	507	53
>5	25	3
Parity		
0-2	281	30
3-5	594	62
Socio demographic characteristics	N=956	
	n	%
Parity		
>5	77	8
Mean (±SD)	3.5(3.1)	
Median (IQR)	3(2-4)	
Undergone VIA test		
Yes	249	26
No	707	74

**Table 2:** Association between Women’s Socio Demographic Characteristics and VIA Screening, Keraniganj Upazila and Tejgaon Area of Dhaka District, Bangladesh, 2017

Socio demographic characteristics				
	Undergo VIA test, n (%)	Number of respondents (N)	OR (95% CI)	P value
Age (Years)				
≤ 35	86 (26)	336	Ref	
36-40	54 (26)	209	1.01 (0.68 - 1.50)	0.95
41-45	49 (37)	132	1.72 (1.12 -2.64)	0.014
46-50	36 (25)	142	0.99 (0.63 -1.55)	0.956
51-55	16 (23)	70	0.86 (0.47 -1.58)	0.631
56-60	7 (16)	44	0.54 (0.24 -1.28)	0.165
> 60	1 (4)	23	0.13 (0.02 -1.00)	0.049
Residence				
Urban	119 (29)	441	Ref	
Semi Urban	130 (24)	545	0.77 (0.58 -1.03)	0.076
Marital status				
Married	226 (26.8)	845	Ref	
Others	23 (21)	111	0.72 (0.44 -1.16)	0.176
Educational status				
Illiterate/only Signature	51 (23)	230	Ref	
Primary	188 (27)	696	1.3 (0.91-1.85)	0.147
Secondary	10 (35)	30	1.75 (0.77-3.99)	0.179
Occupation				
Housewife	239 (26)	928	Ref	
Employee	10 (36)	28	1.6 (0.73 -3.52)	0.241
Monthly expenditure (Tk)				
≤15,000	52 (20)	269	Ref	
16,000-30,000	159 (29)	565	1.63 (1.15 - 2.33)	0.007
31,000-90,000	38 (31)	122	1.89 (1.16 -3.08)	0.011
Religion				
Muslim	242 (26)	931	Ref	
Non-Muslim	7 (28)	25	1.11 (0.46 -2.68)	0.822
Age at first marriage (Years)				
≤15	79 (20)	395	Ref	
16-20	111 (29)	383	1.63 (1.17-2.27)	0.004
21-25	27 (27)	102	1.44 (0.87 - 2.38)	0.156
≥26	32 (43)	76	2.91 (1.73 - 4.88)	0
Parity				
0-2	67 (24)	281	Ref	
3-5	164 (28)	594	1.22 (0.88 - 1.69)	0.238
>5	17 (22)	77	0.9 (0.49 -1.66)	0.746

**Table 3:** Absolute and Relative (%) Number of Women’s Answer Claiming to have Knowledge, Attitude and Practice Regarding Cervical Cancer, Keraniganj Upazila and Tejgaon area of Dhaka district, Bangladesh, 2017

Characteristics	N=956	
	Yes, n	%
Knowledge on female’s common disease		
Cancer	944	98
Cervical cancer	836	87
Leucorrhea	748	78
Dysmenorrhea	401	42
Chronic infection in birth canal	350	36
Breast cancer	337	35
Sexually transmitted disease	164	17
Galactorrhea	33	4
Knowledge on risk factors on Ca Cervix		
Early marriage	527	55
Sexually transmitted disease	401	42
Unhygienic condition of cervix	313	33
Multiple sexual partners	139	15
Conceive more than 5 times	131	14
Human Papilloma Virus	121	13
Long term use of contraceptives > 5 years	105	11
Habit of using tobacco	46	9
Habit of using alcohol or substances	64	7
Knowledge on prevention of Ca cervix		
Early marriage	591	62
Prevention of chronic infection	238	25
HP vaccine use	213	22
Avoid of multiple sexual partners	184	19
Continuous use of condom	133	14
Knowledge on symptoms of CA cervix		
Heaviness in lower abdomen	290	30
Foul smelling vaginal discharge	238	25
Weight loss	228	24
Bleeding in between periods	216	23
Abdominal Pain after intercourse	203	22
Bleeding after intercourse	151	16
Vaginal bleeding after menopause	90	9
Knowledge on test and treatments		
Knowledge of treatment of Ca cervix	618	65
Knowledge of tests for Ca cervix detection	481	50
Cervical cancer vaccine	358	37
Attitude regarding Ca cervix and screening		
Will you advice another women to do a VIA test	883	92
Do you ever recommend a VIA test for all women	870	91
Willingness of nearest female neighbors to get VIA test	597	62
Willing to undergo a VIA test in future	563	59
After knowing risk, will you plan to do VIA test	326	34
Do you think you have any risk for cervical cancer	314	33
Practice regarding Ca cervix and screening		
Did you ever receive HPV vaccine?	18	2
Have you participated in an awareness program regarding Ca cervix and VIA test?	59	6
Have you ever had a VIA test?	249	26
Do you use napkin or clean cloth during menstruation?	632	66
Did you seek medical teatment for female disease?	778	81

**Table 4:** Association between Women’s Socio-Demographic Characteristics and Sufficient Score of Knowledge, Attitude and Practice Regarding Cervical Cancer, Keraniganj Upazila and Tejgaon area of Dhaka district, Bangladesh, 2017

Socio demographic characteristics	Knowledge (n=441)		Attitude (n=365)		Practice (n=181)	
	N (%) with score ≥11 (sufficient knowledge of CC)	OR (L-U)	N (%) with score ≥ 5	OR (L-U)	N (%) with score ≥ 3	OR (L-U)
Age (Years)						
≤ 35	173 (52)	Ref	159 (48)	Ref	63 (19)	Ref
36-40	104 (50)	0.93 (0.66-1.32)	89 (43)	0.93 (0.66-1.32)	41 (20)	0.83 (0.58-1.17)
41-45	63(48)	0.86 (0.57-1.29)	50 (34)	0.86 (0.57-1.29)	38 (29)	0.68 (0.45-1.02)
46-50	55 (39)	0.6 (0.4-0.89)	41 (29)	0.6 (0.4-0.89)	20 (14)	0.45 (0.3-0.69)
51-55	24 (34)	0.49 (0.29-0.84)	16 (30)	0.49 (0.29-0.84)	13 (19)	0.33 (0.18-0.6)
56-60	13 (30)	0.4 (0.2-0.78)	8 (18)	0.4 (0.2-0.78)	5 (11)	0.25 (0.11-0.55)
>60	9 (39)	0.61 (0.26-1.44)	2 (9)	0.61 (0.26-1.44)	1 (4)	0.11 (0.02-0.46)
Marital status						
Married	402 (48)	Ref	345 (41)	Ref	163 (19)	Ref
Others	39 (35)	0.6 (0.4-0.9)	20 (18)	0.6 (0.4-0.9)	18 (16)	0.32 (0.19-0.53)
Residence						
Urban	202 (49)	Ref	105 (25)	Ref	70 (17)	Ref
Semi urban	239 (44)	0.81 (0.62-1.04)	260 (47)	0.81 (0.62-1.04)	111 (20)	2.66 (2.01-3.51)
Educational status						
Illiterate/only Signature	73 (32)	Ref	75 (33)	Ref	34 (15)	Ref
Primary	347 (50)	2.14 (1.56-2.93)	280 (40)	2.14 (1.56-2.93)	141 (20)	1.39 (1.02-1.91)
Secondary	21 (70)	5.02 (2.19-11.49)	10 (33)	5.02 (2.19-11.49)	6 (20)	1.03 (0.46-2.32)
Occupation						
Housewife	423 (46)	Ref	354 (38)	Ref	175 (20)	Ref
Employee	18 (64)	2.15 (0.98-4.71)	11 (39)	2.15 (0.98-4.71)	6 (21)	1.05 (0.49-2.27)
Monthly expenditure (Tk)						
≤ 15,000	107 (40)	Ref	124 (46)	Ref	42 (16)	Ref ()
16,000-30,000	257 (46)	1.26 (0.94-1.7)	200 (35)	1.26 (0.94-1.7)	115 (20)	0.64 (0.48-0.86)
31,000-90,000	77 (63)	2.59 (1.67-4.03)	41 (34)	2.59 (1.67-4.03)	24 (20)	0.59 (0.38-0.92)
Religion						
Muslim	424 (46)	Ref	353 (38)	Ref	178 (19)	Ref
Non-Muslim	17 (68)	2.54 (1.09-5.95)	12 (48)	2.54 (1.09-5.95)	3 (12)	1.51 (0.68-3.35)
Age at first marriage (Years)						
≤15	157 (40)	Ref	133 (34)	Ref	54 (14)	Ref
16-20	193 (50)	1.54(1.16-2.05)	148 (39)	1.54(1.16-2.05)	87 (23)	1.24 (0.93-1.66)
21-25	55 (54)	1.77(1.14-2.75)	47 (46)	1.77(1.14-2.75)	22 (22)	1.68 (1.08-2.62)
≥26	36 (47)	1.36(0.83-2.23)	37 (49)	1.36(0.83-2.23)	18 (24)	1.87 (1.14-3.07)
Parity						
0-2	148 (54)	Ref	109 (39)	Ref	48 (17)	Ref
3-5	268 (45)	0.74 (0.56-0.98)	231 (40)	0.74 (0.56-0.98)	125 (21)	1 (0.75-1.34)
>5	23 (30)	0.38 (0.22-0.66)	23 (30)	0.38 (0.22-0.66)	7 (9)	0.67 (0.39-1.16)

**Table 5:** Association between Undergone VIA Screening and Score of Knowledge and Attitude, Keraniganj Upazila and Tejgaon area of Dhaka district, Bangladesh, 2017

Scores	Undergo VIA-test, n (%)	Crude OR	95% CI	Adjusted OR	95% CI
Score of knowledge					
Insufficient	77 (15)	Ref		Ref	
Sufficient	172 (39)	3.64	2.67-4.95	2.48	1.56-2.83
Score of attitude					
Insufficient	145 (24)	Ref			
Sufficient	104 (28)	1.22	0.91-1.64		

**Table 6:** Source of Information Regarding Cervical Cancer, Keraniganj Upazila and Tejgaon area of Dhaka district, Bangladesh, 2017

Source of information	n	%
Source of information regarding cervical cancer		
Friends/ neighbors’/relatives	634	76
Television	423	51
Doctor	398	48
Health workers	197	26
Campaign	93	11
Nurse	63	8
Non-governmental organization	43	5
Internet	28	4
Radio	24	3
Source of information regarding cervical cancer screening test		
Friends/Relatives/close Acquaintances’	290	60
Doctor	243	50
Television	168	35
Health Workers	97	20
Any Campaign	53	11
Nurse	38	8
Internet	19	4
NGO	11	2
Radio	11	2

## Discussion

Early detection is essential to prevent cervical cancer. In Bangladesh, visual inspection with the acetic acid test is commonly used to detect cancer and helps in preventing deaths due to cervical cancer. Despite its effectiveness as a method of early detection and controlling cervical cancer incidence, our study showed significant underutilization of VIA screening test. According to Health Bulletin 2019, only 7.5% women aged 30 to 49 years in Bangladesh age group had gone under cervical screening test in their lifetime where our study showed that among 956 women, 26% of the participants in Dhaka District ever performed the VIA test. This increased difference is due to an ongoing cervical cancer prevention program in Dhaka District where we conducted this study. Another study in Bangladesh showed that only 300,000 women gone through VIA screening and a low coverage rate was observed during the cervical cancer screening program (Basu et al., 2010). In most studies, socio demographic factors of the participants such as age, income, and age at first marriage showed a positive association with the VIA test. The most represented age group of our study population was ≤ 35 years which similar the study held in Cameroon (Agarwal et al., 2012). Our study findings showed that women of 41-45 years old were more likely to test VIA than the youngest group because cervical cancer tends to occur during midlife (Cervical Cancer Overview-NCCC,”n.d). This is consistent with a study conducted for developing countries (Dhamija et al., 1993). As expected, women living in urban areas in Bangladesh are more likely to have a VIA test than women living in the semi urban area. Woldetsadik et al. also showed that women who were living in semi urban areas in country were less likely to be screened than women who were living in the urban area (Woldetsadik et al., 2020).

The results revealed that 87% of participants knew about cervical cancer which is consistent with the study done in Bangladesh (Rahman and Bhattacharjee, 2019; Islam et al., 2015). This result is also similar to a study conducted in Northern India where 92% of participants heard of cervical cancer (10) and in Congo it is 82% which is close to our study (Ali-Risasi et al., 2014). Touch et al. reported 74% of study women living in Kampong Speu, Cambodia, had ever heard about cervical cancer (Touch and Oh, 2018). Shrestha et al. said 66% had heard about cervical cancer (Shrestha et al., 2013). In our study, friends, neighbors, and relatives (76%) were the primary information sources for cervical cancer and screening tests for the respondents. These results are congruent with those reported by Gupta et al., (2019). In a study done in Hosanna, Ethiopia, 10% of participants had VIA test for cervical cancer and 2% of the respondents had taken vaccine (Aweke et al.,2017). which is lower than reported by Gupta et al., (2019) where 5% of their participants had undertaken the HPV vaccination (Gupta et al.,2019) and 6% of them ever participated in any awareness program. 

In our study, females knew about cervical cancer and recommended other women to get a VIA test but few women got a VIA test. In Bangladesh VIA screening is free and available in most medical college hospitals, Upazila health complex and maternal health and NGO clinics. Any women aged 30 years or above can request a VIA test. Underutilization of VIA test may occur because women may be shy as the exam rooms are usually not private and they don’t know anything about that VIA is a screening test for cervical cancer. The test is free, but most women think they have to pay for the test. Women have less confidence about the test because nurses instead of a doctor usually performs the VIA test. Women sometimes face social stigma if they test positive. In the author’s experience, doctors prefer the Pap’s smear over the VIA. 

The major risk factors of cervical cancer are early marriage, sexually transmitted diseases, unhygienic conditions of cervix, and multiple sexual partners (Papri et al., 2015). Lack of awareness of these risk factors was one of the main causes of not having a VIA test. More than half of our study participants knew about early marriage as a risk factor for cervical cancer. This result is in agreement with a study in North West Ethiopia (Mengesha et al., 2020). Only a few of our participants (12%) knew that HPV could be a risk factor for cervical cancer which is similar to a study in North West, Ethiopia (Mengesha et al.,2020). However, in studies in South African and Iraq in which, 49% and 37% of the respondents knew that HPV causes cervical cancer, respectively (Hoque and Hoque, 2009; Haseeb Hwaid, 2013). Women knew about cervical cancer but few of them knew that HPV vaccine could prevent cervical cancer. This could result in lower rate of vaccination to HPV. 

Proper knowledge about cervical cancer and about its symptoms can reduce burden of this disease. If women don’t know about the symptoms of cervical cancer, they will not seek treatment for those symptoms and thus cervical cancer may not be detected at the primary stage. Our study findings showed that respondents have less knowledge about the symptoms of cervical cancer. In our study 33% women thought that they might have risk of cervical cancer and respondents had lowest knowledge about sexually transmitted disease which should be increased to prevent cervical cancer caused by sexual transmissions. Among the study population 62% women knew that cervical cancer could be prevented by putting a stop to early marriage. More than one-fifth of the participants believed that HPV vaccine could prevent cervical cancer. It is similar in agreement with a study of Bangladesh (Islam et al., 2018). Half of our participants knew about the availability of screening tests for cervical cancer. It is consistent with this study of North West Ethiopia (Mengesha et al., 2020). But it is much lower than another study conducted in a tertiary hospital in India, where 85% of the women knew about screening tests for cervical cancer (Varadheswari et al., 2015). Nearly sixty-five percent of the participants knew that treatment could cure cervical cancer. It is much higher than the study conducted in North West Ethiopia, where only 28% knew that cervical cancer is curable (Mengesha et al., 2020). This study showed that only 38% knew about the cervical cancer vaccine, which was very low. It is similar to this study, where 36% of the women knew about the preventive vaccine (Gupta et al., 2019).

Education has played a critical role in enhancing our participant’s knowledge of cervical cancer. Cervical cancer knowledge is likely to increase gradually in women with secondary education than women with no education. A study in the Maldives showed knowledge improved with literacy level improvement (Basu et al., 2014). Dhamija et al. also brought out that literate women had better awareness about cervical cancer and related information (Dhamija et al., 1993). In another study in Ethiopia, literate women were more likely to be knowledgeable by 22.7 times than women who were illiterate (Kassa et al., 2019). The knowledge of testing VIA was more likely in employed women than housewives, but it was not statistically significant. Papri et al. showed that the knowledge of cervical cancer screening was significantly higher among the employed women. Emphasizing on higher education cervical can also be held back.

When women have greater attitude towards VIA screening then the trend of testing VIA will increase. The percentage of attitude among the majority of the respondents was high on parameters like willingness to do VIA tests in the future, advising anyone to do a VIA test. However, the attitude was low regarding other parameters. For instance, 33% believed in having the risk of cervical cancer, and even after knowing the risk, only 34% would plan to do a VIA test. In contrast, Narayan et al. reported that most of the respondents showed a positive attitude towards cervical cancer (Narayana et al., 2017). The VIA test attitude was 2.5 times higher in the respondents of semi-urban areas than the urban area. Married women showed three times higher attitude regarding the VIA test than divorced, widowed, or separated women. It seems that the likelihood of attitude towards VIA test increases as the age at first marriage increases. Education may play a role in preventing early age at first marriage. 

With the help of UNFPA and Bangabandhu Sheikh Mujib Medical University (BSMMU), the Government of Bangladesh (GOV) started cervical cancer screening in all districts of Bangladesh. This screening program aims to test the women who are at risk though many of them may have no symptoms of the diseases. In developing countries though there are different screening program are ongoing, but these programs fail to meet their target screening of cervical cancer because of social and financial deficiency (Sankaranarayan et al., 2001). When the screening program will be well organized people will be more interested to participate in screening. Making the test more reliable to the women in root level can also increase the practice of testing VIA.

This study found that, although most participants knew about cervical cancer and screening tests, the percentage of VIA screening tests was low and practice of taking vaccine was very poor. The reason behind having low vaccine and less screening might be not being financially solvent, lack of logistics and social obstacles. Education, employment, residence, income plays a vital role in increasing knowledge, attitude, and the practice of cervical cancer screening test. This study reveals a significant relationship between a sufficient score of experience and undergoing a VIA test. Women who have sufficient knowledge are more likely to test VIA than those who have insufficient knowledge.

In conclusion, this study found that, although most participants knew about cervical cancer and screening tests, the percentage of VIA screening tests is low and practice of taking vaccine is very poor. Education, employment, residence, income plays a vital role in increasing knowledge, attitude, and the practice of cervical cancer screening test. This study reveals a significant relationship between a sufficient score of experience and undergoing a VIA test. Women who have sufficient knowledge are more likely to test VIA than those who have insufficient knowledge.

*Limitations*

This study had several limitations. Study participants were reluctant to answer sensitive questions which may bias results of associations. We recruited study subjects from two communities and are not representative of the country. Our study recruited women 30 years and above, thus our results cannot be interpreted for younger women. Study population did not include women from remote areas because of difficult terrain and lack of security for data collectors. Thus, conclusions may not represent all women in the communities we conduct this study. We did not conduct focus group discussions which would obtain more details on the knowledge, attitude and beliefs regarding cervical cancer and VIA screening. 

*Recommendations*

Women need to know that VIA is a screening for cervical cancer and it is free to increase utilization of VIA. Attitude women are more confident when doctors perform the test rather than nurse. So if doctors can be rostered or counsel patient about testing procedure with privacy and confidentiality testing VIA would increase simultaneously. Expansion of awareness program regarding cervical cancer and giving more effort in changing attitude can influence the practice of having vaccine and undergoing VIA screening positively. 

## Author Contribution Statement

Md. Omar Qayum: conception, protocol writing, field activities and data collection, monitoring, supervision, analysis, manuscript writing and review, Mallick Masum Billah: analysis and manuscript review, Rehena Akhter: manuscript review, Meerjady Sabrina Flora: protocol writing, analysis and manuscript review.
